# SiO_2_ NPs-PQ/PMMA Photopolymer Material Doped with a High-Concentration Photosensitizer for Holographic Storage

**DOI:** 10.3390/polym12040816

**Published:** 2020-04-04

**Authors:** Ying Liu, Fenglan Fan, Xiaodi Tan

**Affiliations:** 1 Research Institute for Frontier Science, Beihang University, No. 37th, Xueyuan Road, Haidian District, Beijing 100091, China; 2 Research Center for Quantum Sensing, Zhejiang Lab, No. 1818th, Wenyi West Road, Yuhang District, Hangzhou 310000, Zhejiang, China; 3 Department of Chemistry and Chemical Engineering, Hebei Normal Universitry for Nationalities, Chengde 067000, Hebei, China; ffl619@163.com; 4 Fujian Provincial Key Laboratory of Photonics Technology, College of Photonic and Electronic Engineering, Fujian Normal University, Fuzhou 350007, Fujian, China

**Keywords:** nanocomposite photopolymer material, holographic data storage, polarization holography, holographic storage material

## Abstract

Dispersing nanoparticles and increasing the photosensitizer concentration have been regarded as effective approaches for improving the performance of a holographic storage material. In this paper, SiO_2_ nanoparticle (NP)-dispersed PQ/PMMA nanocomposite material (SiO_2_ NP-PQ/PMMA) with a high PQ doping concentration was prepared. By introducing the co-monomer methyl isobutyl ketone (MIBK) that comes from an SiO_2_ NP colloidal solution, the concentration of PQ in the system increased to 1.2 wt %. We investigated the performance of polarization holographic recordings in both traditional PQ/PMMA and nanocomposite material SiO_2_ NP-PQ/PMMA with the orthogonally polarized signal and reference waves. With the dispersion of the SiO_2_ NPs colloidal solution and the increase in the PQ concentration, diffraction efficiency and photoinduced birefringence were multiplied. In addition, high-quality holographic image reconstruction was achieved by our homemade material.

## 1. Introduction

Holographic storage material is a key factor restricting the development of holographic storage technology. The storage capacity, data transmission rate, storage life, and stability of a holographic storage device are closely related to the performance of the recording material [1]. PQ (phenanthrenequinone) is sensitive to light and often used as a holographic storage material photosensitizer. PQ/PMMA (phenanthrenequinone-doped poly(methyl methacrylate)) photopolymer has polarization sensitivity, controllable thickness, good optical quality, and low shrinkage (contraction coefficient < 10^−5^), all of which make it suitable as a holographic storage material for multidimensional storage [2,3,4]. However, due to the low PQ saturation concentration dissolved in MMA and to the poor photosensitivity associated with a limited quantum of PQ molecular reactions [5], PQ/PMMA is unsatisfactory regarding diffraction efficiency and polarization sensitivity.

In general, there are two main ways to increase the number of reactions of PQ molecules. One is to increase the amount of PQ molecules reacted by adding nano-components to introduce the interdiffusion of nanoparticles and PQ molecules. In 2001, Vaia R. A. et al. [6] first incorporated gold nanoparticles into photopolymers. Suzuki N. et al. [7,8,9] incorporated a series of non-metal oxides (SiO_2_, TiO_2_, and ZrO_2_) into the methacrylate organic photopolymer system. It was found that, by adjusting the blending ratio, the refractive index modulation of the material could be improved, and the incorporation of nanoparticles helps to improve the stability of the material and reduce the shrinkage rate [10,11,12,13]. The research of the doping system has unique research value.

The other is to directly increase the concentration of the photosensitizer. Since the solubility of PQ in an MMA solution at room temperature is only 0.7 wt %, H. Liu et al. [14] reported a method for increasing the PQ concentration by increasing the prepolymerization temperature. The results showed that about 1.0 wt % PQ was dissolved in the MMA solution at a reaction temperature of 60 °C. Mahilny et al. [15] demonstrated that the concentration of PQ can be increased to 4 mol % by casting the liquid solution directly onto the substrate and drying it to a solid state, but the method is only suitable for relatively low thickness (50–180 μm). Recently, Fenglan Fan et al. [16,17] proposed chemically modifying the material components to prepare a photopolymer material with PQ-loaded co-monomer, which improved the solubility of the photoinitiator in the photopolymer and thus promoted the optical properties of the material. 

In this paper, a kind of SiO_2_ nanoparticle-dispersed PQ/PMMA composite holographic storage material (SiO_2_ NP-PQ/PMMA) containing a high photosensitizer PQ concentration was prepared. Photosensitizer PQ has certain solubility in an MIBK solution (a protective solution of doped SiO_2_ nanoparticles), which is considered a photopolymer co-monomer. The addition of an MIBK solution with a certain solubility to photosensitizer PQ increases the PQ concentration (to 1.2 wt %) in photopolymer materials. The influence of the newly introduced solution on the reaction of the system and the improvement of the performance of the holographic storage material were analyzed. In addition, a series of experiments were carried out on the prepared SiO_2_ NP-PQ/PMMA nanocomposites for the material reaction and holographic diffraction characteristics.

## 2. Material Preparation 

**Figure 1:**
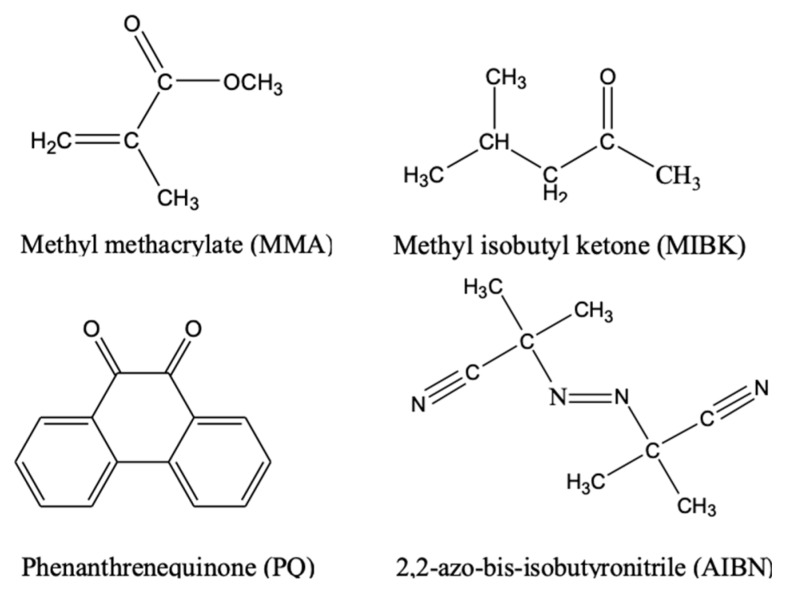
Chemical structures of the major components in the photopolymer sample.

Figure 1 shows chemical structures of the major components in the photopolymer sample. MMA and methyl isobutyl ketone (MIBK, from an SiO_2_ NP colloidal solution) were regarded as the co-monomers, PQ was used as the photosensitizer, and 2,2-azobisisobutyronitrile (AIBN) was employed as the thermo-initiator. The SiO_2_ NP colloidal solution contains 30% SiO_2_, 69.5% MIBK, and 0.5% CH_4_O. The size of each SiO_2_ NP is about 20 nm. In this paper, the SiO_2_ NP colloidal solution contained the nano-doped components.

In our fabricating process, since PQ molecules have solubility in both the MMA monomer and MIBK, an introduced SiO_2_ NP colloidal solution can increase the dissolved concentration of the PQ molecule. The monomer MMA and SiO_2_ NPs were mixed firstly with a weight ratio of 100:3. After weighing, the SiO_2_ NP colloidal solution, the monomer MMA, the photosensitizer PQ, and the thermal initiator AIBN were mixed in a clean reaction bottle at a certain mass ratio in a dark room at room temperature. The proportion of each component in the mass ratio of MMA is shown in Table 1 (SiO_2_ NPs/MIBK = 3:7).

Since the viscosity of MIBK dopants is higher than that of the MMA solution, excessive SiO_2_ NP colloidal solution will affect the copolymerization, leading to many small bubbles inside the material. Photopolymer samples with an SiO_2_ NP doping concentration of 3 wt % were chosen.

The sample battle was ultrasonically shaken in an ultrasonic cleaner to form a uniform multi-component solution. Subsequently, the homogeneously mixed solution was placed in a magnetically stirred, constant-temperature water bath. The temperature was continuously raised to 60 °C to start the prepolymerization process and was stabilized at 60 °C for an appropriate period until the homogeneous solution became viscous (glycerol viscosity). The viscous solution was poured into a specific glass mold. The mold was placed in a blast oven at 60 °C for about 40 h, until the material was completely cured. Finally, the mold was removed and placed in a refrigerator for 2 h. The prepared SiO2 NP-PQ/PMMA material was bulk with millimeter-level dimensions. It had good optical transparency, and the color was light yellow. As the concentration of photosensitizer and material thickness increased, the sample color deepened.

## 3. Results

### 3.1. UV–Vis Spectra Measurements

**Figure 2:**
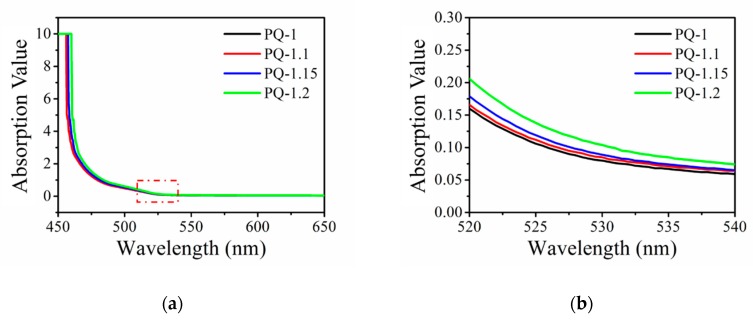
(**a**) UV–visible absorption spectrum of SiO_2_ NP-PQ/PMMA sample; (**b**) partially amplified UV–Vis absorption spectrum.

The optical absorption of the material had an important impact on the holographic performance. According to Beer’s law, the absorbance *A* is proportional to the product of concentration c of the light-absorbing substance and the optical path length b of the absorption cell (*A* = *ε•c•b*). For the UV–Vis spectroscopic technique, absorption can be indicated as *A* = −*lg* (*I_T_*/*I_0_*), in which *I_0_* is the intensity of the incident light entering the substance and *I_t_* is the intensity of the transmitted light emitted from the back substance. In order to determine the effect of the introduced SiO_2_ NP colloidal solution and the high concentration PQ on the optical absorption of the prepared SiO_2_ NP-PQ/PMMA material, optical absorption was measured using a TU-1901 dual-beam UV–Vis spectrophotometer (PERSEE, Beijing, China) at 25 °C. The thickness of the sample was 1.5 mm. The plot of absorbance versus the wavelength is shown in Figure 2. The absorption trend of the SiO_2_ NP-PQ/PMMA sample shows no obvious change in the absorption curve of the conventional PQ/PMMA photopolymer material. The absorption in the short-wave direction was strong and substantially zero when λ > 600 nm, shown in Figure 2a. A red laser with a wavelength of 632.8 nm (which did not cause a change in the optical properties of the material) was used as the probe beam for the experiment with a refractive index modulation change caused by the photoinduced anisotropy of the material. As depicted in Figure 2b, the sample has a certain absorption at 532 nm, and by increasing PQ concentration, the absorption value of the material has little change at 532 nm. Thus, in the subsequent holographic recording experiments, a green laser of 532 nm was selected as the recording light to induce an anisotropic reaction in the sample material.

### 3.2. FT-IR Spectra Measurements

**Figure 3:**
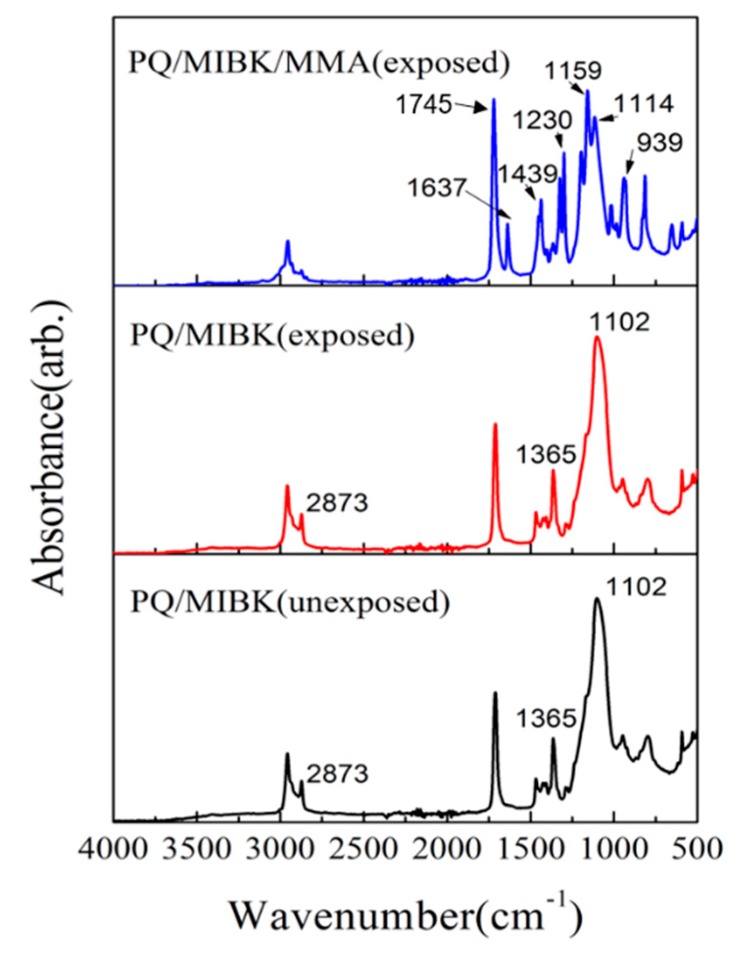
FT-IR absorption spectra of unexposed PQ/MIBK, exposed PQ/MIBK, and exposed PQ/MIBK/MMA.

Infrared spectroscopy can obtain photochemistry reaction information about the samples. We measured the FT-IR absorption spectrum of PQ and the monomers before and after photo-irradiation to investigate whether new photoproducts were formed by adding the SiO_2_ NP colloidal solution. Figure 3 shows the FT-IR spectra of unexposed and exposed PQ/SiO_2_ NP colloidal solution (bottom, middle of Figure 3) and exposed PQ/SiO_2_ NP colloidal solution/MMA samples (top of Figure 3), which were obtained with a Nicolet 6700 Infrared Spectrometer (Thermo Fisher Scientific Inc., Waltham, MA, USA). Since the main component of the doped SiO_2_ NP colloidal solution was MIBK, we considered MIBK as a co-monomer.

The FT-IR spectra of the PQ/MIBK solution before and after exposure are mostly the same; no obvious new bond was formed. We suggest that there was no photopolymerization reaction between the MIBK solution and the PQ molecules. The main photoproduct was still formed by the reaction of MMA molecules and PQ molecules. Within the broad absorption of 2800–3000 cm^−1^, all samples had a broad absorption, which was derived from the C–H bond stretching (CH_3_ and CH_2_ stretching). A strong absorption peak at about 1745 cm^−1^ resulted from a carbonyl group commonly contained in the MIBK and MMA units. The absorption band at about 1230 and 939cm^−1^ was mainly due to the C–O–C group of the photoproduct formed by the reaction of the group in the PQ molecule and the vinyl group in the monomer molecule. These phenomena indicate that the added MIBK solution had no effect on the photochemical reaction of the photosensitizer PQ molecule or of the monomeric MMA molecule.

### 3.3. Holographic Diffraction Characteristics

Photosensitizer PQ molecules possess a high conjugate coplanar structure [18]. Before the illumination, PQ molecules with different orientations were randomly distributed in the material. When the material is exposed by a linearly polarized wave, dependent on the polarization state, PQ molecules with a certain orientation have a greater chance of reacting with monomers on certain regions [5,19]. The orientation of the PQ molecules that was parallel to the illuminated wave polarization state had a greater chance of reacting. Double bonds in the carbonyl functional group of PQ molecules were excited by illuminated photons. PQ molecules became radicals. PQ radicals reacted with MMA monomers that had carbonic double bonds on the vinyl functional group. The photoproduct finally formed [20,21]. This photoreaction can cause the polarization distribution. More photosensitizer PQ molecules can participate in the reaction when more PQ molecules are doped within a certain range. Thus, the concentration of photosensitizer PQ plays an important role on polarization holographic recording. Furthermore, a mutual diffusion process occurred in the SiO_2_ NP-PQ/PMMA photopolymer sample, in which the dispersed NPs played a positive role in enhancing the diffraction efficiency formation [22]. Along with the consumption of PQ molecules and the formation of photoproducts, the embedded SiO_2_ NPs introduced a multicomponent diffusion process. As a result, the SiO_2_ NP composition gradually increased the amount of PQ molecules participating in the reaction, and the amount of photoproduct increased correspondingly. Therefore, the addition of the SiO_2_ NP colloidal solution improved the saturated diffraction efficiency of the material.

**Figure 4:**
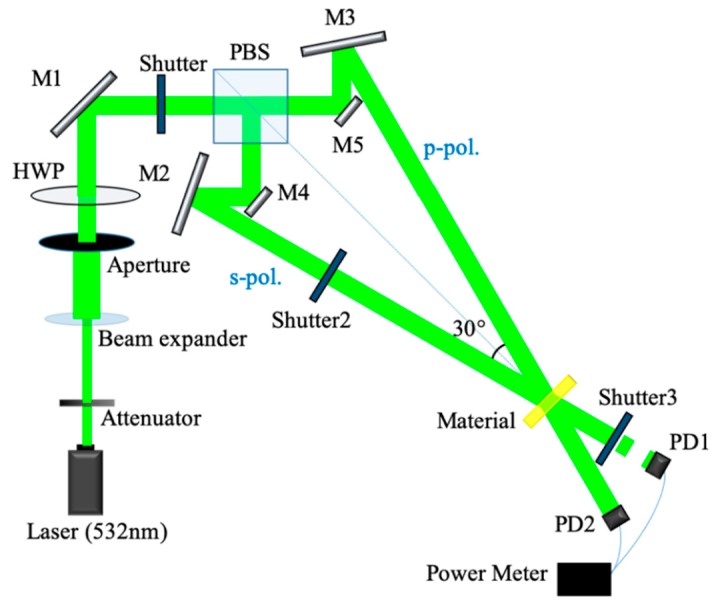
Experimental setup for holographic diffraction efficiency measurement, where HWP: half-wave plate; PBS: polarization beam splitter; PD: photo detector; M: mirror; s-pol.: vertical polarization light; p-pol.: horizontally polarized light.

Figure 4 shows the experimental setup of the holographic diffraction characteristic measurements. In the experiment, the collimated laser (532 nm), from a diode-pumped solid-state Nd:YAG laser, was split into a vertically polarized wave (s-pol., signal wave) and a horizontally polarized wave (p-pol., reference wave) by a polarization beam splitter (PBS). We recorded diffraction gratings by two orthogonal linearly polarized waves. They were incident to the material symmetrically and received by two photodetectors.

In the recording stage, the volume polarization hologram was written at a cross-angle of 30°, and each beam intensity was 22 mW. The shutters were used to control the holographic recording time. The recording process and the reconstruction process were separated after each period of exposure. In the recording process, Shutters 1 and 2 were opened for 4 s, while Shutter 3 was closed. In the reconstruction process, Shutter 2 closed and Shutter 3 opened. The original reference wave irradiated the material to retrieve the grating for 0.4 s. At this moment, we obtained the corresponding diffraction signal after the 4 s recording. The recording process and the reconstruction process were carried out on a continuous loop until the power of reconstructed wave was saturated.

**Figure 5:**
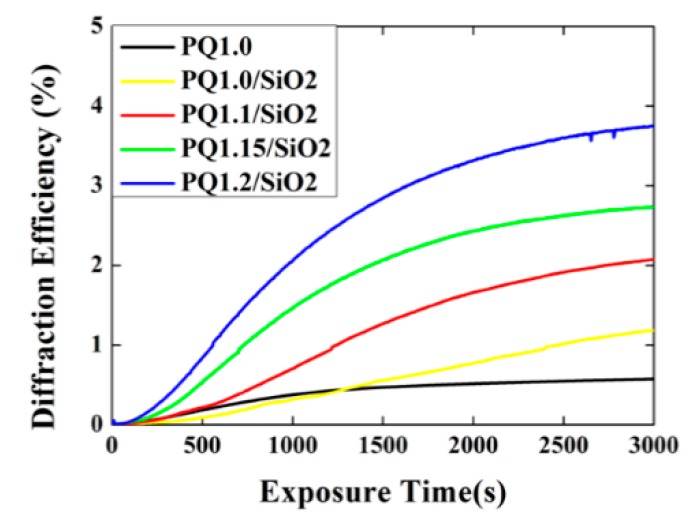
The dependence of the orthogonal linearly-grating diffraction efficiency on exposure time.

**Figure 6:**
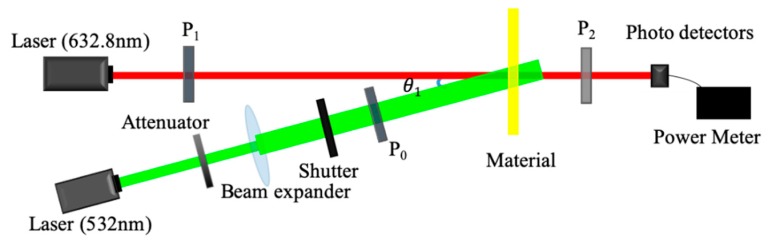
Diagram of photoinduced birefringence measurement device: *θ* = 6°, P_0_, P_1_, and P_2_ are horizontal polarizers, negative 45° polarizers and positive 45° polarizers, respectively.

Figure 5 shows the temporal evolution of the orthogonal linearly-grating diffraction efficiency for the SiO_2_ NP-PQ/PMMA samples containing different photosensitizer concentrations. The diffraction efficiency *η* is defined as I_+1_/ (I_0_ + I_+1_) in this paper, and I_0_ and I_+1_ are the intensities of the transmitted and the 1st-order diffracted wave, respectively. In Figure 6, PQ1.0, PQ1/SiO_2_, PQ1.1/SiO_2_, PQ1.15/SiO_2_, and PQ1.2/SiO_2_ indicate Sample 2, Sample 3, Sample 4, Sample 5, and Sample 6, respectively. The saturated diffraction efficiency of the recorded grating was improved compared with the undoped sample (PQ1.0). With the increase in PQ concentration, the saturation diffraction efficiency increased correspondingly. The saturation diffraction efficiency was increased to nearly 4%. The diffraction efficiency of PQ1.0 increased faster than that of PQ1/SiO_2_, at low exposure times. This is because the presence of MIBK monomers caused a decrease of MMA monomers in the same region, while PQ monomers did not increase. Hence, compared with PQ1.0, fewer PQ molecules were involved in the reaction for PQ1/SiO_2_ at low exposure times. Subsequently, due to the continuous consumption of PQ, the effect of dynamic redistribution from NPs was obvious. More PQ molecules were involved in the reaction, which caused the diffraction efficiency to increase.

From the above experimental analysis, it can be deemed that the concentration of photosensitizer is important for photopolymer materials and that nanoparticle doping can improve the performance of the material. It is feasible to use a nanoparticle protection solution to increase the concentration of photosensitizer and form a two-monomer composite system to improve the holographic properties of the material. The concentration of the photosensitizer PQ in the PQ/PMMA photopolymer system increases based on the introduction of favorable nano-components, and the material is further optimized.

### 3.4. Photoinduced Birefringence

Photoinduced birefringence *Δn* is one of the key parameters of polarization holographic material. For a PQ/PMMA material system, photoinduced birefringence is mainly caused by the structural rearrangements induced by the photochemical reaction of PQ molecules [18,23]. The photoinduced birefringence is given by (ignore the absorption) [24]:

(1) $$\Delta n=n_{1}-n_{2}=\frac{\lambda }{\pi d}arcsin\sqrt{\frac{I_{T}}{I_{0}sin^{2}2\theta_{0}}}$$

where *n*_1_, *n*_2_, *d*, λ, *I*_0_, *I_T_*, and *θ_0_* are the refractive index along and perpendicular to the polarization direction of the pump light, the thickness of the photopolymer sample, the wavelength of the pumping laser, the intensity of the probe wave before pumping exposure, the intensity of the probe wave after pumping exposure, and the angle between the polarization direction of the green laser and that of the red laser, respectively.

We explored the photoinduced birefringence of the photopolymer sample pumped by a diode pumped solid state (DPSS) Nd:YAG laser (λ = 532 nm). The experimental setup is schematically shown in Figure 6. The photoinduced birefringence was investigated with a continuous He-Ne laser (λ = 632.8 nm) as the probe light, which is far from the photopolymer absorption band. In the experimental detection, the intensity of the pumping wave incident on the sample surface was 20mW controlled by the attenuator, and the diameter of the spot was 6 mm; the power of the probe light incident on the material surface after passing through Polarizer 1 was 0.8 mW, and the diameter of the spot was 2 mm. The cross-angle *θ*_1_ between the two beams was set to 6°. Firstly, the photopolymer was illuminated by a separate probe wave, and no transmitted wave was received. The simple was isotropic. After the linearly-polarized pumping wave was turned on, the intensity of the transmitted probe wave gradually increased. The sample developed into anisotropy by the oriented photoproduct molecules in the sample.

**Figure 7:**
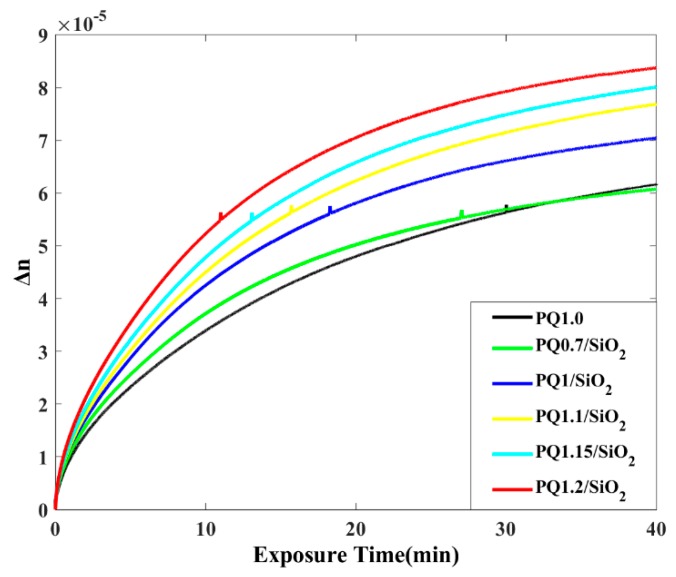
Temporal evolution of photoinduced birefringence.

Figure 7 shows the temporal evolution of the sample photoinduced birefringence, in which PQ0.7/SiO_2_ indicates Sample 1. At the initial stage of pumping exposure, the photoinduced birefringence of the sample increased rapidly with the cumulative exposure energy, and PQ molecules formed double-substituted phenanthrene photoproducts. The photoinduced birefringence slowly changed after a certain period of time, when most of the PQ molecules, whose directions are the same or similar to that of the polarized light field, have absorbed the energy and when photolysis has occurred. With the same composition of dispersed SiO_2_ NPs in the sample, accompanied by an increased PQ molecule concentration, the material achieved a high Δn value. In addition, the doping of nano-components also enhanced the photoinduced birefringence of the material. When doped with nanoparticles, the photoinduced birefringence curve of the sample with a 0.7 wt % PQ concentration was similar to that of the sample with a 1.0 wt % PQ concentration without nanoparticles doping.

### 3.5. Application Experiments

**Figure 8:**
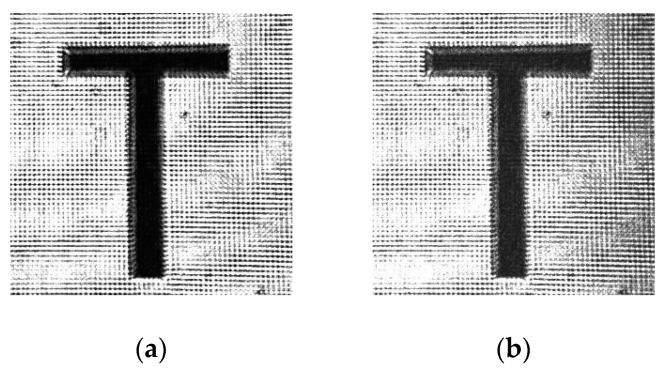
Image reconstruction results in polarization holography: (**a**) original transmitted image; (**b**) reconstructed image.

We recorded and reconstructed a real image on the SiO_2_ NP-PQ/PMMA photopolymers by our polarization holographic recording system [17] to better exhibit the material application of the polarization holography. In the experiment, the signal wave and reference wave of polarization holographic recording were s-polarized and p-polarized, respectively. The image was recorded in a sample (MMA/SiO_2_ NPs/PQ = 100:3:1.2) approximately 10 × 10 × 15 mm. The original image for polarization holographic recording was uploaded onto spatial light modulators (SLM). Figure 8a shows the original transmitted image that was directly transmitted and transformed to a digital signal by a picture receiver (CMOS). Figure 8b shows the reconstructed image that was recorded in the SiO_2_ NP-PQ/PMMA sample by a polarization holographic recording. Both images were 300 × 300 pixels. The reconstructed image was reconstructed faithfully and had a clear contrast and high fidelity. The information polarization recording competence of the SiO_2_ NP-PQ/PMMA material was shown. The feasibility and potential of our material for recording polarization multiplexed holograms was thus demonstrated.

## 4. Conclusions

In this work, we provided an effective pathway to overcoming the PQ solubility limitation and to simultaneously introducing an NP modulation of PQ/PMMA. The introduction of an SiO_2_ NP colloidal solution increased the concentration of PQ to 1.2 wt %. The newly introduced solution caused no reaction in the system. Compared with the undoped PQ/PMMA material, the saturated diffraction efficiency of the grating formed by SiO_2_ NP-PQ/PMMA nanocomposites under a polarized hologram recorded by orthogonal linear polarized light increased from 0.6% to nearly 4%. It is thus shown that controlling the doping of nanoparticles and the concentration of the photosensitizer can improve the performance of holographic photopolymer storage material. Therefore, a solution with higher compatibility can be selected as a composite component of the system to improve the performance of holographic photopolymer storage materials.

## Figures and Tables

**Table 1:** Concentration ratio of each component in the prepared sample.

Sample	MMA (wt %)	SiO_2_ (wt %)	PQ (wt %)	AIBN (wt %)
1	100	3	0.7	1
2		0	1	
3		3	1	
4		3	1.1	
5		3	1.15	
6		3	1.2	
